# Supplementary material for: Wood stock in neotropical streams: Quantifying and comparing instream wood among biomes and regions
Source: PLoS One. 2022 Oct 5;17(10):e0275464. doi: 10.1371/journal.pone.0275464 (PMC9534444; doi:10.1371/journal.pone.0275464)
Supplement: S1 Table — (DOCX) [file pone.0275464.s001.docx]

**S1. Table.** **Channel and catchment measurements taken in field and spatial assessments.**

|  | **METRIC** | **CÓD.** | **UNIT** |
| --- | --- | --- | --- |
| 1. | Reach length | REACHLEN | m |
| 2. | Wetted width | XWIDTH | m |
| 3. | Bankfull width | XBKF_W | m |
| 4. | Bankfull height | XBKF_H | m |
| 5. | Thalweg depth | XDEPTH_T | cm |
| 6. | Mean cross–section depth | XDEPTH_CS | cm |
| 7. | Bankfull thalweg depth^a^ | BKF_DEPTH | m |
| 8. | Bankfull cross-section area^b^ | BKF_AREA_CS | m² |
| 9. | Bankfull planform area^c^ | BKF_AREA_PF | m² |
| 10. | Channel slope | XSLOPE_% | % |
| 11. | Sinuosity | SINU | – |
| 12. | Bed material type | SUBSTRATE | – |
| 13. | Substrate size | DGM | mm |
| 14. | Flow type | FLOW | – |
| 15. | Mean residual depth | RP100 | cm |
| 16. | Sum of 3 Riparian forest vegetation areal cover on banks (visual evaluation) | XCMGW | Areal proportion– |
| 17. | Large wood count per reach length | C1W_100 | pieces/100m |
| 18. | Large wood count per channel area | C1W_100MSQ | pieces/100m² |
| 19. | Large wood volume per reach length | V1W_100 | m³/100m |
| 20. | Large wood volume per channel area | V1W_100MSQ | m³/100m² |
| 21 | Average length of large wood pieces | LW_LENGTH | m |
| 22 | Average diameter of large wood pieces | LW_DIAM | m |
| 23. | Catchment mean elevation | CAT_ELEV | m |
| 24. | Catchment mean slope | CAT_SLO | % |
| 25. | Catchment area | CAT_AREA | Km² |
| 26. | Catchment forest cover | CAT_FOR | % |
| 27. | Riparian forest cover in the upstream network within 100m buffer | NET_FOR | % |
| 28. | Riparian forest cover within 100m buffer along the study reach | LOC_FOR | % |

^a^ Bankfull height + thalweg depth

^b^ Cross section depth * bankfull width

^c^ Bankfull width * reach length
