# Supplementary material for: Wood stock in neotropical streams: Quantifying and comparing instream wood among biomes and regions
Source: PLoS One. 2022 Oct 5;17(10):e0275464. doi: 10.1371/journal.pone.0275464 (PMC9534444; doi:10.1371/journal.pone.0275464)
Supplement: S2 Table — Mean, standard deviation and range are presented. (DOCX) [file pone.0275464.s002.docx]

**S2. Table. Amounts and dimensions of LW in the six Brazilian study regions.** Mean, standard deviation and range are presented.

| **Biome** | **Amazon**  Mean ± SD (range) | | | **Cerrado**  Mean ± SD (range) | | | | | **Both**  Mean ± SD (range) |
| --- | --- | --- | --- | --- | --- | --- | --- | --- | --- |
| **Region**  **Metric** | **PGM** | **STM** | **Sub-total** | **SS** | **NP** | **TM** | **VG** | **Sub-total** | **Total** |
| LW pieces  (nº) | 37.63 ± 36.16  (0 – 127) | 25.63 ± 17.33  (0 – 81) | 31.81 ± 29.11  (0 – 127) | 41.08 ± 35.02  (0 – 138) | 20.23 ± 17.89  (0 – 69) | 32.25 ± 42.54  (0 – 169) | 41.13 ± 47.81  (0 – 193) | 33.62 ± 38.19  (0 – 193) | 32.93 ± 34.94  (0 – 193) |
| LW volume  (m³) | 6.60 ± 6.77  (0 – 24.26) | 3.60 ± 5.04  (0 – 31.16) | 5.15 ± 6.15  (0 – 31.16) | 8.70 ± 14.81  (0.00 – 73.96) | 4.18 ± 4.74  (0.00 – 24.34) | 11.14 ± 20.00  (0.00 – 106.94) | 7.51 ± 11.76  (0.00 – 53.94) | 7.88 ± 14.05  (0.00 – 106.94) | 6.83 ± 11.73  (0.00 – 106.94) |
| LW diameter  (m) | 0.26 ± 0.04  (0.20 – 0.34) | 0.23 ± 0.03  (0.20 – 0.33) | 0.24 ± 0.04  (0.20 – 0.34) | 0.26 ± 0.05  (0.20 – 0.37) | 0.26 ± 0.05  (0.20 – 0.33) | 0.28 ± 0.07  (0.20 – 0.44) | 0.25 ± 0.06  (0.20 – 0.45) | 0.26 ± 0.06  (0.20 – 0.45) | 0.26 ± 0.05  (0.20 – 0.45) |
| LW length  (m) | 3.93 ± 0.59  (3.25 – 5.64) | 3.68 ± 0.52 (3.25 – 5.46) | 3.80 ± 0.57  (3.25 – 5.64) | 3.94 ± 0.68  (3.25 – 6.21) | 4.07 ± 0.69  (3.25 – 5.74) | 4.37 ± 1.19  (3.25 – 7.90) | 3.92 ± 0.79 (3.25 – 6.88) | 4.07 ± 0.87  (3.25 – 7.90) | 3.97 ± 0.78  (3.25 – 7.90) |
| LW abundance  (nº/100m) | 25.08 ± 24.10  (0.00 – 84.67) | 17.08 ± 11.55  (0.00 – 54.00) | 21.21 ± 19.41  (0.00 – 84.67) | 24.70 ± 21.78  (0.00 – 91.33) | 13.19 ± 11.96  (0.00 – 46.00) | 18.48 ± 24.42  (0.00 – 93.33) | 25.68 ± 31.69  (0.00 – 127.33) | 20.49 ± 23.88  (0.00 – 127.33) | 20.76 ± 22.23  (0.00 – 127.33 |
| LW abundance  (nº/100m²) | 4.54 ± 4.51  (0.00 – 0.16) | 1.63 ± 1.60  (0.00 – 0.07) | 3.13 ± 3.71  (0.00 – 0.16) | 5.25 ± 6.87  (0.00 – 0.28) | 2.77 ± 3.00  (0.00 – 0.16) | 3.93 ± 5.35  (0.00 – 0.18) | 6.18 ± 8.98  (0.00 – 0.34) | 4.53 ± 6.50  (0.00 – 0.34) | 3.99 ± 5.63  (0.00 – 0.34) |
| LW load  (m³/100m) | 4.40 ± 4.52  (0.00 – 16.18) | 2.40 ± 3.36  (0.00 – 20.77) | 3.43 ± 4.10  (0.00 – 20.77) | 4.71 ± 6.92  (0.00 – 30.72) | 2.55 ± 2.62  (0.00 – 12.17) | 6.82 ± 13.11  (0.00 – 71.29) | 4.67 ± 7.52  (0.00 – 35.96) | 4.69 ± 8.48  (0.00 – 71.29) | 4.21 ± 7.14  (0.00 – 71.29) |
| LW load  (m³/100m²) | 0.84 ± 0.93  (0.00 – 3.39) | 0.23 ± 0.35  (0.00 – 1.73) | 0.55 ± 0.77  (0.00 – 3.39) | 1.05 ± 2.07  (0.00 – 11.42) | 0.49 ± 0.45  (0.00 – 1.68) | 1.63 ± 3.83  (0.00 – 21.32) | 1.08 ± 2.29  (0.00 – 14.17) | 1.06 ± 2.48  (0.00 – 14.17) | 0.86 ± 2.02  (0.00 – 21.32) |
| LW diam./ channel depth | 0.24 ± 0.08  0.12 – 0.16) | 0.31± 0.13  0.12 – 0.72) | 0.28 ± 0.11  (0.12 – 0.72) | 0.18 ± 0.05  (0.11 – 0.34) | 0.24 ± 0.08  (0.12 – 0.46) | 0.22 ± 0.07  (0.11 – 0.34) | 0.21 ± 0.09  (0.10 – 0.54) | 0.21 ± 0.08  (0.10 – 0.54) | 0.24 ± 0.10  (0.10 – 0.72) |
| LW length/ channel width | 0.73 ± 0.33  (0.08 – 1.66) | 0.42 ± 0.36  (0.03 – 1.91) | 0.57 ± 0.38  (0.03 – 1.91 | 0.70 ± 0.39  (0.18 – 2.01) | 0.85 ± 0.35  (0.27 – 1.61) | 0.91 ± 0.61  (0.26 – 2.45) | 0.86 ± 0.73  (0.27 – 4.75) | 0.83 ± 0.54  (0.18 – 4.75) | 0.73 ± 0.50  (0.03 – 4.75) |
