# Supplementary material for: Wood stock in neotropical streams: Quantifying and comparing instream wood among biomes and regions
Source: PLoS One. 2022 Oct 5;17(10):e0275464. doi: 10.1371/journal.pone.0275464 (PMC9534444; doi:10.1371/journal.pone.0275464)
Supplement: S3 Table — Mean, standard deviation and range are presented. (DOCX) [file pone.0275464.s003.docx]

**S3. Table. Catchment and channel characteristics of the streams belonging to the six Brazilian studied regions.** Mean, standard deviation and range are presented.

| **Biome** | **Amazon**  Mean ± SD (range) | | | **Cerrado**  Mean ± SD (range) | | | | | | |
| --- | --- | --- | --- | --- | --- | --- | --- | --- | --- | --- |
| **Region**  **Metric** | **PGM** | | **STM** | **SS** | **NP** | | **TM** | | | **VG** |
| Catchment characteristics | | | | | | | | | | |
| Area (Km²) | | 12.55 ± 12.39  (0.44 – 50.37) | 28.70 ± 47.07  (0.83 – 227.13) | 30.23 ± 26.93  (0.37 – 108.45) | | 10.74 ± 10.70  (1.38 – 50.74) | | 45.23 ± 47.21  (0.45 – 164.97) | 27.53 ± 30.22  (2.64 – 116.43) | |
| Catchment slope (%) | | 4.64 ± 1.86  (1.55 – 9.49) | 7.22 ± 2.95  (3.96 – 14.80) | 5.59 ± 1.81  (3.10 – 9.65) | | 8.24 ± 3.03  (3.16 – 17.16) | | 7.36 ± 3.22  (3.40 – 16.72) | 5.94 ± 1.85  (3.21 – 12.74) | |
| Catchment forest cover (%) | | 81.13 ± 18.68  (35.39 – 100.00) | 90.43 ± 8.03  (65.38 – 100.00) | 12.99 ± 6.35  (0.81 – 27.37) | | 36.57 ± 24.98  (7.84 – 99.19) | | 45.57 ± 18.03  (14.78 – 100.00) | 11.56 ± 5.32  (0.10 – 22.80) | |
| Network forest cover (%) | | 78.76 ± 20.38  (23.84 – 100) | 88.26 ± 11.4  (51.23 – 100) | 35.78 ± 18.11  (0.00 – 100) | | 62.33 ± 20.13  (25.29 – 100) | | 55.75 ± 18.41  (23.21 – 100) | 33.08 ± 12.91  (1.39 – 61.71) | |
| Channel characteristics | | | | | | | | | | |
| Bankfull width (m) | 8.00 ± 7.26  (2.86 – 39.73) | | 17.33 ± 18.24  (1.70 – 100) | 6.92 ± 3.22  (1.87 – 18.71) | 5.45 ± 2.31  (2.13 – 14.06) | | 6.52 ± 3.59  (1.48 – 13.99) | | | 6.00 ± 2.70  (0.94 – 11.87) |
| Bankfull depth (m) | 1.10 ± 0.26  (0.56 – 1.71) | | 0.85 ± 0.31  (0.34 – 1.74) | 1.50 ± 0.36  (0.85 – 2.71) | 1.18 ± 0.33  (0.54 – 1.92) | | 1.34 ± 0.38  (0.74 – 2.36) | | | 1.33 ± 0.36  (0.54 – 2.08) |
| Bankfull width/ bankfull depth (w/d) | 7.93 ± 8.09  (2.68 – 38.62) | | 19.10 ± 15.77  (3.15 – 85.95) | 4.67 ± 2.08  (1.50 – 12.42) | 5.01 ± 3.23  (1.47 – 21.70) | | 5.02 ± 2.94  (1.77 – 13.84) | | | 4.50 ± 1.70  (1.75 – 9.18) |
| Channel slope (%) | 0.32 ± 0.27 (0.02 – 1.60) | | 0.67 ± 0.69  (0.06 – 3.07) | 0.81 ± 0.56  (0.05 – 2.64) | 1.35 ± 0.84  (0.39 – 4.76) | | 0.60 ± 0.59  (0.01 – 2.38) | | | 0.82 ± 1.22  (0.07 – 6.86) |
| Bankfull discharge (m³/s) | 5.02 ± 5.28 (0.42 – 28.33) | | 8.42 ± 10.64  (0.12 – 42.20) | 29.38 ± 26.70  2.17 – 105.00) | 25.18 ± 57.74  (0.88 – 360.19) | | 9.76 ± 10.05  (0.63 – 43.44) | | | 16.92 ± 21.22  (0.17 – 106.21) |
| Stream power (W/m) | 242.14 ± 630.49  (0.87 – 4,441.77) | | 429.78 ± 573.48  (6.75 – 2,452.60) | 2960.58 ± 4895.76  (41.15 – 27,131.23) | 3190.61 ± 7032.15  (87.62 – 43,181.52) | | 707.72 ± 1145.51  (1.56 – 4,944.90) | | | 2145.38 ± 5595.36  (12.72 – 29,771.34) |
| Hydraulic resistance | 0.09 ± 0.089  (0.02 – 0.50) | | 0.17 ± 0.11  (0.00 – 0.16) | 0.02 ± 0.02  (0.00 – 0.09) | 0.03 ± 0.03  (0.00 – 0.50) | | 0.08 ± 0.07  (0.01 – 0.34) | | | 0.04 ± 0.05  (0.00 – 0.24) |
| Substrate size (mm) | 0.70 ± 1.79  (0.01 – 12.69) | | 1.45 ± 8.12  (0.01 – 56.45) | 29.91 ± 126.51  (0.01 – 780.90) | 8.69 ± 21.42  (0.02 – 129.78) | | 168.34 ± 590.46  (0.01 – 3,613.40) | | | 72.81 ± 335.15  (0.01 – 2,113.95) |
| Woody riparian forest (%) | 77.72 ± 56.02  (3.86 – 231.14) | | 126.11 ± 54.93  (9.55 – 213.98) | 65.55 ± 41.80  (0.00 – 170.57) | 62.60 ± 30.90  (3.41 – 130.11) | | 52.87 ± 32.63 (1.59 – 175.34) | | | 67.98 ± 42.44  (4.32 – 174.43) |
| Local forest cover (%) | 62.11 ± 35.36  (0.00 – 100.00) | | 70.60 ± 28.80  (1.79 – 100.00) | 31.92 ± 26.91  (0.00 – 92.86) | 63.47 ± 29.85  (6.67 – 100.00) | | 49.51 ± 29.35  (0.00 – 100.00) | | | 40.01 ± 25.88  (0.00 – 100.00) |
