# Supplementary material for: Wood stock in neotropical streams: Quantifying and comparing instream wood among biomes and regions
Source: PLoS One. 2022 Oct 5;17(10):e0275464. doi: 10.1371/journal.pone.0275464 (PMC9534444; doi:10.1371/journal.pone.0275464)
Supplement: S4 Table — The world biomes were classified following Trimble & van Aarde [69]. (DOCX) [file pone.0275464.s004.docx]

**S4. Table. Large wood assessments in streams around the world according to biome.** The world biomes were classified following Trimble & van Aarde [69].

| **Biome** | **Study** | **LW abund.**  **n/100m** | **LW volume**  **m³/100m** | **LW volume**  **m³/100m²** | **LW length**  **(m)** | **LW diameter**  **(m)** | **Channel width**  **(m)** | **Slope**  **(%)** |
| --- | --- | --- | --- | --- | --- | --- | --- | --- |
| Boreal forest / Taiga | Dahlstrom & Nilsson (2004) | 51.00 | - | 0.59 | 2.60 | 0.10 | 1.95 | 0.06 |
|  | Kreutzweiser et al. (2005) | 19.25 | - | - | - | 0.17 | 4.93 | 0.02 |
|  | Mossop & Bradford (2004) | 29.12 | - | 0.34 | 3.97 | 0.15 | 5.20 | 0.02 |
|  | Robison & Beschta (1990) | 33.40 | 58.00 | 6.08 | 7.40 | 0.53 | 11.40 | 0.02 |
| Temperate Broadleaf and mixed forest | Cordova et al. (2007) | 9.83 | - | 0.93 | - | - | 5.46 | 0.01 |
|  | Diez et al. (2001) | - | - | 0.66 | - | - | 5.34^a^ | 0.09 |
|  | Iroumé et al. (2014) | - | - | 4.52 | - | - | 9.91 | 0.07 |
|  | Meleason et al. (2005) | 38.64 | 17.97 | 2.06 | - | - | 3.79 | 0.05 |
|  | Warren & Kraft (2008) | 34.94 | 3.53 | - | - | - | - | - |
|  | Webb & Erskine (2005) | 64.50 | - | 4.08 | - | - | - | - |
| Temp. Conifer Forest/ Temp. Broadleaf Forest | Deng et al. (2002) | 11.68 | - | 1.72 | - | - | - | - |
|  | Seo & Nakamura (2009) | - | - | 0.04 | - | - | 19.12 | 0.10 |
| Temperate Conifer Forest | Comiti et al. (2006) | 21.20 | 1.90 | 0.40 | 2.54 | 0.14 | - | 0.16 |
|  | Fox & Bolton (2007) | 57.02 | 54.57 | - | - | - | - | - |
|  | May & Gresswell (2003) | 40.25 | - | - | - | - | 3.82 | 0.14 |
|  | Nowakowski & Wohl (2008) | - | 10.72 | 0.43 | 2.74 | 0.14 | 5.12* | - |
|  | Reeves et al. (20003) | 15.91 | 150.14 | - | - | - | - | - |
|  | Wallace & Benke (1984) | - | 35.60 | 1.58 | - | - | 4.92 | - |
|  | Zelt & Wohl (2004) | 62.00 | 28.07 | 20.50 | 6.90 | 0.21 | 10.05 | 0.02 |
| Tropical and Subtropical Moist Broadleaf Forest | Cadol et al. (2009) | 77.07 | 17.03 | 12.33 | 3.95 | 0.19 | 7.44 | 0.02 |
|  | Paula et al. (2013) | 1.31 | - | 0.09 | 4.25 | 0.17 | 2.21* | 0.12 |
|  | Saraiva et al. (present study) | 21.21 | 3.43 | 0.55 | 3.80 | 0.24 | 12.52 | 0.00 |
| Tropical Forest/ Savanna | Saraiva et al. (present study) | 24.70 | 4.71 | 1.05 | 3.94 | 0.26 | 6.92 | 0.01 |
| Savanna | Pettit et al. (2005) | - | - | 0.90 | - | - | 277.52 | - |
|  | Saraiva et al. (present study) | 19.12 | 4.68 | 1.07 | 4.11 | 0.27 | 5.99 | 0.01 |

^a^ Wetted channel width
