# Supplementary material for: Wood stock in neotropical streams: Quantifying and comparing instream wood among biomes and regions
Source: PLoS One. 2022 Oct 5;17(10):e0275464. doi: 10.1371/journal.pone.0275464 (PMC9534444; doi:10.1371/journal.pone.0275464)
Supplement: Attachment — Submitted filename: Response to Reviewers.docx [file pone.0275464.s006.docx]

27-Aug-2022

To: Luiz Ubiratan Hepp, Academic Editor, PLOS ONE

Re: PLOS ONE manuscript [PONE-D-22-12647] - [EMID:6ef87f8211645570]: “Wood stock in neotropical streams: quantifying and comparing in-stream wood among biomes and regions”

Dear Dr Hepp

We have revised our manuscript in response to the reviewers’ comments and your recommendations. We thank the reviewers for their insights, evaluation, and suggestions on the presentation of our research, and believe that the revised manuscript is much improved and look forward to you considering it again for acceptance for publication in PLOS ONE.

The main issue pointed out by both reviewers was the lack of clarity about the importance of studying wood in aquatic ecosystems, and how our research was related to our objectives and hypotheses. In response, we improved wording, especially in the introduction, adding more information and references about the importance of wood in rivers and streams, since this subject is well established in the Riverine Science literature (Lines 58-73). Reviewer #1 had a number of valuable suggestions and comments concerning the relevance of the research and tying the results and discussion more tightly to our objectives and hypotheses stated in the Introduction. We complied with all those suggestions and responded to each one individually (see below). All changes can also be seen on the attached marked-up copy of our revised manuscript.

We did not present sufficient justification in our draft manuscript to convince Reviewer #2 of the relevance of our research. We believe that the additional information in our revision will clarify for this reviewer and other readers the importance of our research on wood stocks in flowing waters. On two points we express our disagreement with this reviewer’s opinion:

1) Wood in streams is inherently relevant to aquatic ecology, geomorphology, and management. Note that whole symposia have been focused on this subject. “The International Conference Wood in World Rivers” is held every four years and brings together riverine researchers from around the world specifically to discuss this relevant topic. This segment of Riverine Science was inaugurated in the 1970’s (Swanson *et al.*, 2020) by pioneer researchers like Frederick Swanson from the Pacific Northwest in USA, and since then, studies on this topic have been spreading to the rest of the world (Ruiz-Villanueva & Stoffel, 2017). After 50 years of research, there is no doubt about the importance of wood to aquatic systems. However, there is a consensus on the need for more studies on in-stream wood to expand knowledge to regions not yet studied, especially in face of deforestation and degradation of the riparian corridors around the world (Wohl, 2017; Wohl *et al.*, 2019). Therefore, this is currently a topic that is especially relevant for the tropics.
2) Simply characterizing ecologically important aspects of aquatic resources in an under-studied region is indeed relevant to ecology and management. The physical processes in fluvial environments are a dominant driver affecting biota directly and indirectly. Numerous studies have already shown how the physical habitat has crucial role in affecting biological communities (Casatti *et al.*, 2006; Zeni & Casatti, 2014; Castro *et al.*, 2018), instream wood included (Herdrich *et al.*, 2018; Leitão *et al.*, 2018; Sterling & Warren, 2018). The natural wood regime is recognized as the third leg of the tripod of physical processes, together with the hydrological regime and sediment flow (Wohl *et al.*, 2019). We agree that research on the relationship of wood and related aspects of physical habitat to aquatic biota is certainly valuable. However, we strongly disagree with the argument that we should have included in this manuscript the analysis of macroinvertebrates or any other biological group to demonstrate the importance of studying wood in rivers. Actually, we do not think that emphasis on relevance of wood to macroinvertebrates is necessary or desirable. The importance of wood (in its own right) to channel morphology, sediment transport, streambed stability, hydrologic retention, enduring pools, flood flow refugia, carbon storage, and habitat complexity is overwhelming. Wood is extremely important to all these things, and in turn, those things are important to macroinvertebrates, fish, benthic algae, processing of nutrients, and ecosystem services to humans. Detailed consideration of macroinvertebrates is too narrow a focus for this article and would dilute and complicate the exposition of our research on wood in streams.

Following and in the marked-up draft of our manuscript, we show our response to detailed comments provided by Reviewer #1:

R**esponse to Reviewer #1:**

Specific details

Introduction

Line 54 delivery or input? Considering that the wood stock (branches, logs and roots) falls from the trees, the term input may be more appropriate.

*Changed.*

Line 68 adds references to temperate regions.

*Added.*

Line 70 (and in every manuscript) biome or bioclimatic regions? What do the authors consider biomes in the study (differences in wood stock)? What is the regions approach?

*Biome definition included. Use of the terms more consistently.*

Line 73 environments or biomes?

*Here we used the word "environments" because we are saying in general. It is important to study different biomes, bioclimatic regions, ecosystems and wherever, to really understand how the wood regime works and its variation on the space.*

Line 99 – 101 repeated information

*We deleted the repeated sentence.*

Line 99 – 105 confused

*Wording improved.*

Line 111 - I did not find any hypothesis associated with this objective (iii) and the introduction is not directed to this topic.

*We rewrote the objectives.*

Material and Methods

Lines 238-246, associated with H2 (or a new hypothesis?)

*Associated with H2.*

Lines 247 – 259, associated with H2, but with insertion of new categories. These terms should be cited in the introduction. Or the MM section should be better structured: two studies.

*We changed the way of presenting information from USA streams. Now we present it as part of our study, but only with comparative purposes.*

Results

Line 271 Brazilian streams, regions in the Amazonia and Cerrado were evaluated.

*Changed.*

Lines 297-303, results of Hypothesis 1, comparison between regions.

*Changed.*

Lines 348-382 I did not find the hypothesis associated with these results.

*Solved.*

Discussion

Line 559, Brazilian biomes, Amazonia and Cerrado biomes.

*Changed.*

Lines 559- 648 I did not find the hypothesis associated with these discussion.

*Solved.*

**References:**

Casatti, L., Langeani, F. & Ferreira, C.P. (2006) Effects of physical habitat degradation on the stream fish assemblage structure in a pasture region. *Environmental Management*, **38**, 974–982.

Castro, D.M.P. de, Dolédec, S. & Callisto, M. (2018) Land cover disturbance homogenizes aquatic insect functional structure in neotropical savanna streams. *Ecological Indicators*, **84**, 573–582.

Herdrich, A.T., Winkelman, D.L., Venarsky, M.P., Walters, D.M. & Wohl, E. (2018) The loss of large wood affects rocky mountain trout populations. *Ecology of Freshwater Fish*, **27**, 1023–1036.

Leitão, R.P., Zuanon, J., Mouillot, D., Leal, C.G., Hughes, R.M., Kaufmann, P.R., Villéger, S., Pompeu, P.S., Kasper, D., de Paula, F.R., Ferraz, S.F.B. & Gardner, T.A. (2018) Disentangling the pathways of land use impacts on the functional structure of fish assemblages in Amazon streams. *Ecography*, **41**, 219–232.

Ruiz-Villanueva, V. & Stoffel, M. (2017) Frederick J. Swanson’s 1976–1979 papers on the effects of instream wood on fluvial processes and instream wood management. *Progress in Physical Geography*, **41**, 124–133.

Sterling, K.A. & Warren, M.L. (2018) Effects of introduced small wood in a degraded stream on fish community and functional diversity. *Southeastern Naturalist*, **17**, 74–94.

Swanson, F.J., Gregory, S. V., Iroumé, A., Ruiz‐Villanueva, V. & Wohl, E. (2020) Reflections on the history of research on large wood in rivers. *Earth Surface Processes and Landforms*, esp.4814.

Wohl, E. (2017) Bridging the gaps: An overview of wood across time and space in diverse rivers. *Geomorphology*, **279**, 3–26.

Wohl, E., Kramer, N., Ruiz-Villanueva, V., Scott, D.N., Comiti, F., Gurnell, A.M., Piegay, H., Lininger, K.B., Jaeger, K.L., Walters, D.M. & Fausch, K.D. (2019) The natural wood regime in rivers. *BioScience*, **69**, 259–273.

Zeni, J.O. & Casatti, L. (2014) The influence of habitat homogenization on the trophic structure of fish fauna in tropical streams. 259–270.
